# Polylactic Acid/Polycaprolactone Blends: On the Path to Circular Economy, Substituting Single-Use Commodity Plastic Products

**DOI:** 10.3390/ma13112655

**Published:** 2020-06-10

**Authors:** Marc Delgado-Aguilar, Rita Puig, Ilija Sazdovski, Pere Fullana-i-Palmer

**Affiliations:** 1 ABBU Research Group, Department of Computer Science and Industrial Engineering, Universitat de Lleida (UdL), Pla de la Massa 8, 08700 Igualada, Spain; rita.puig@udl.cat; 2 UNESCO Chair in Life Cycle and Climate Change ESCI-UPF, Universitat Pompeu Fabra, Passeig Pujades 1, 08003 Barcelona, Spain; ilija.sazdovski@esci.upf.edu (I.S.); pere.fullana@esci.upf.edu (P.F.-i-P.)

**Keywords:** polymer blends, polycaprolactone, polylactic acid, micromechanical analysis, mechanical properties

## Abstract

Circular economy comes to break the linear resource to waste economy, by introducing different strategies, two of them being: using material from renewable sources and producing biodegradable products. The present work aims at developing polylactic acid (PLA), typically made from fermented plant starch, and polycaprolactone (PCL) blends, a biodegradable polyester, to study their potential to be used as substitutes of oil-based commodity plastics. For this, PLA/PCL blends were compounded in a batch and lab scale internal mixer and processed by means of injection molding. Tensile and impact characteristics were determined and compared to different thermoplastic materials, such as polypropylene, high density polyethylene, polystyrene, and others. It has been found that the incorporation of PCL into a PLA matrix can lead to materials in the range of 18.25 to 63.13 megapascals of tensile strength, 0.56 to 3.82 gigapascals of Young’s modulus, 12.65 to 3.27 percent of strain at maximum strength, and 35 to 2 kJ/m^2^ of notched impact strength. The evolution of the tensile strength fitted the Voigt and Reuss model, while Young’s modulus was successfully described by the rule of mixtures. Toughness of PLA was significantly improved with the incorporation of PCL, significantly increasing the energy required to fracture the specimens. Blends containing more than 20 wt% of PCL did not break when unnotched specimens were tested. Overall, it was found that the obtained PLA/PCL blends can constitute a strong and environmentally friendly alternative to oil-based commodity materials.

## 1. Introduction

Plastic or plastic materials are the terms used to describe a huge family of very different materials with singular properties, characteristics, and thus, applications. They can either be fossil-based or bio-based, and in a few cases, they can biodegrade. The plastic consumption worldwide is increasing day by day since its introduction into the market during the first half of the last century. Plastics play an important role in our economy. In fact, according to Plastics Europe, the plastic industry gives direct employment to over 1.5 million people in about 60,000 industries in Europe, which had a turnover close to 355 billion euros in 2017. The same year, from the 348 million tons that were produced worldwide, 64.4 million tons were produced in Europe, most of them fossil-based, for several market sectors, such as packaging (39.7%), building and construction (19.8%), automotive (10.1%), and others [1].

While there is no doubt that plastics are crucial for maintaining our modern daily life, they can have serious downsides on the environment and health [2]. According to Geyer et al. (2017), about 302 tons of plastic waste was generated in the world in 2015, considering both primary and secondary plastics. Assuming that, between 1950 and 2015, approximately 12 percent of this plastic waste has been typically incinerated and 9 percent has been recycled, it comes to the light that the rest of the plastic waste (79%, 238.6 tons in 2015) was directly landfilled or accumulated in the natural environment [3]. In addition to this, conventional plastics such as polypropylene (PP), polyethylene (PE), polystyrene (PS), polyvinylchloride (PVC), polyethylene terephthalate (PET), or even polyester, polyamide, and acrylic (PP&A) fibers, are usually produced from fossil fuels and are non-biodegradable in nature [4]. Further, most of these non-biodegradable polymers that reach the environment through littering, when exposed to sunlight and moisture, usually experience fragmentation into particles that may reach millimeters or even micrometers in size, also known as microplastics [5]. The presence of such plastic residues in the environment has negative impacts on the environment, such as alterations of the food chain, groundwater pollution, air pollution, and other unknown effects, especially those derived from microplastics [6,7]. Some may argue that including mineral functional fillers within the polymers decreases the amount of plastic producing impact, it is clear that this end of life impact of plastic will be diminished if they are biodegradable [8].

For all the above, in addition to adequate domestic waste management that can trigger significant benefits for the environment, other strategies at upper levels must be implemented, such as extended producer responsibility (EPR) and adequate governmental strategies and policies aiming at the transition from a linear economy to a circular bioeconomy [9,10,11,12]. Indeed, several efforts are being paid to achieve an independent economy and society from non-renewable resources. The use of greener materials such as bio-based and biodegradable plastics and composites as commodity plastic substitutes, is a clear example [13,14,15,16,17]. As an example, the European Commission (EC) is actively involved on this matter, supporting several actions and projects, as well as defining strategic lines to follow. In a recent document, the need of improving the plastic recycling processes in Europe, decreasing the landfilling areas and promoting the development of plastics recycling was revealed [2]. This is part of the European strategic transition towards a circular bioeconomy and also contributes to the achievement of the Sustainable Development Goals, described by the General Assembly of the United Nations in *The 2030 Agenda for Sustainable Development* [18]. The abovementioned document calls into question the use of oxo-degradable polymers, mainly due to its disintegration in marine environments and its negative effects on marine flora and fauna, but surprisingly does not make any mention to bio-based or biodegradable polymers. The intentions of the EC against plastic waste are clear in its banning and/or limitation of single-use plastics (SUP), as declared in the Directive (EU) 2019/904 [19], although sometimes may be misleading [20]. Although most of the population is concerned about the negative impacts of plastics in the environment, the responsibility cannot be focused only on the customers, and producers must transform their processes in order to provide more environmentally friendly goods and products. Therefore, the use of bio-based and biodegradable materials and compounds must be encouraged and evaluated thoroughly [21], preferentially through life cycle assessment methodology [22].

The interest on the development of biopolymers started in the early 1930s, with the synthesis of polyhydroxybutyrate (PHB) or high molecular weight polylactic acid (PLA), reported in 1925 and 1932, respectively, among others [23,24]. PLA is a thermoplastic, high-strength and modulus polymer that exhibits good grease and oil resistance and belongs to the family of aliphatic polyesters, which are considered biodegradable and compostable [25,26,27,28]. It can be produced from renewable resources for a wide range of applications, including packaging, semi structural applications, and even medical devices. More recently, PLA has been extensively used as feedstock material in desktop fused deposition modelling filament for 3D printers [29,30]. One of the main advantages is that no special equipment is required to process PLA and it can be easily processed by means of injection molding, extrusion, film blowing, thermopressing, and others [31,32,33,34,35]. Similarly to many other thermoplastic materials, PLA exhibits glass transition and melting temperature, T_g_ and T_m_, respectively. It is well known that the T_m_ of PLA is around 180 °C, which is a relatively low melting temperature, considering that PP, PS, Nylon 6,6 or PET exhibit higher T_m_. However, considering this melting point, PLA exhibits a relatively high T_g_ (around 60 °C). Both temperatures can be modified depending on the molecular weight of the polymer, as well as the ratio between L-lactide and D-lactide [35,36,37]. Regarding mechanical properties, PLA presents the main drawback in its inherent brittleness and poor toughness, which limits its introduction in several markets where flexible commodity polymers are broadly used [38,39,40,41].

To overcome PLA limitations, many approaches have been reported, including the incorporation of fillers and reinforcements, copolymerization strategies, or blending with other polymers [23,26,42,43,44,45]. The polymer industry has been working on polymer blends since the nineteenth century by means of different processes, including batch mixers but also continuous mixers such as extruders [46]. There are several studies focused on the development of PLA blends with other polymers, such as chitosan, thermoplastic starch (TPS), polyhydroxybutyrate (PHB), polycaprolactone (PCL), poly(butylene adipate-co-terephthalate) (PBAT), or even polyamide 11 (PA11) [44,47,48,49,50,51,52,53,54,55]. Among these matrices, PCL deserves special attention due to its biodegradable character and interesting properties in combination with PLA. In addition, PCL is extensively used in biomedical applications for long-term implants and controlled drug release applications [56,57,58]. PCL is a nontoxic, easily obtainable, biocompatible, and biodegradable, but non-bio-based, synthetic polymer [59]. It exhibits a low melting temperature (around 60 °C) and significantly low tensile strength and modulus, but high strain at break [55]. In addition, PCL exhibits a rubbery amorphous phase at room temperature [60].

Blending PLA with PCL has been a topic of interest since many years. One of the main objectives of PLA/PCL blends is the development of improved toughness materials while maintaining the biodegradability and biocompatibility of both phases. One of the main limitations of PLA and PLA/PCL blends is their mechanical performance at high temperature. The low melting temperature of PCL limits their use in applications where high temperature is required. However, the softening temperature of products prepared from neat PLA or PLA/PCL blends are usually governed by the glass transition temperature of PLA [60]. Melting PLA with PCL may decrease the brittleness of PLA, offering a wider range of potential applications, especially those related with toughness. However, it has been reported that the incorporation of PCL may also have a negative impact on tensile strength [61,62]. Some authors found unexpected behaviors of toughness when no coupling agent was incorporated into the PLA/PCL blends and it was attributed to a weak interphase between both polymers. However, the incorporation or the use of compatibilizers has not been found to provide substantial enhancement (while introducing more potentially impacting material into the environment), calling into question the need of using them and the real benefits of adding a third phase into the system [60,63,64,65].

For all the above, the main aim of the present work is to develop different blends of PLA and PCL to cover a wide range of properties, proposing this blend as potential substitute of different plastic commodities that are currently used in high-volume applications, such as polyolefin and other oil-based and non-biodegradable thermoplastics. Although PLA is controversial for facing the problem related to single-use-plastics, under appropriate conditions it could represent a strong alternative. This wide range of applications may contribute to the finding of competitive alternatives to single-use plastic products, as stated above. In addition, the present work also aims at characterizing the PLA/PCL blends obtained by means of internal mixing without the incorporation of any coupling agent. Overall, the present study offers an industrially feasible solution for several applications with no need of sophisticated modifications to the matrices that conform the resulting blend, with apparently less environmental impact.

## 2. Materials and Methods 

### 2.1. Materials

Polylactic acid (PLA) Ingeos Biopolymer 4043D was supplied by Nature Works LLC (Blair, NE, USA) and polycaprolactone (PCL) Capa^TM^ 6500 was kindly supplied by Perstorp Specialty Chemicals AB (Perstorp, Sweden). Both polymers were used for the development of the hybrid matrix. All the materials were dried at 60 °C until constant weight before being processed in an oven (Labopolis, Alcalá de Henares, Spain). The density of each material was determined by means of a glass pycnometer.

### 2.2. Compounding of the Materials

PLA and PCL blends were produced in an internal mixer Brabender Plastograph^TM^ (Duisburg, Germany), controlled by WINMIX software (v3.0.0., Brabender GmbH, Duisburg, Germany). First of all, PLA was introduced into the mixing chamber at 190 °C and 80 rpm. Then, PCL was introduced into the chamber and both temperature and rotational speed conditions were kept until constant torque was observed. The compositions of PLA/PCL ranged from 100/0 to 0/100 wt%. Volume fraction of PCL (V^PCL^) was calculated from density measurements. In all cases, samples were pelletized in a knife mill (Agrimsa,, Villarobledo, Spain) equipped with a 10-mm mesh at the bottom and kept in an oven (Labopolis, Alcalá de Henares, Spain) at 60 °C to prevent moisture absorption. Table 1 shows the different mass and volume compositions of the obtained blends.

As indicated in Table 1, the samples were named based on the reciprocal percentages of PLA and PCL.

### 2.3. Injection Molding of the Blends

Blends were processed by injection molding using an Aurburg 220 M 350-90U equipment (Aurburg, Loßburg, Germany) equipped with a mold for standard testing specimens, according to ISO 178:2010 and UNE-EN ISO 527-1:2012 for flexural and tensile tests, respectively. The equipment is equipped with five heating zones, whose temperatures were set at 175, 180, 185, 185, and 190 °C, respectively, and the pressure was ranged from 330 to 600 bar, depending on the melt flow index (MFI) of the blends. MFI was determined according to ISO 1133-1:2011 and testing conditions were set at 210 °C and 2.16 kg of weight. Results were expressed as mass flow rate in g/10min.

### 2.4. Mechanical Testing of the Blends

Tensile tests were both carried out in an InstronTM 1122 universal testing machine (Metrotec, Lezo, Spain) equipped with a 5-kN load cell and according to ISO 527-1:2012. The gap between clamps was set at 115 mm with a cross-head testing velocity of 2 mm/min. 

Impact strength was measured according to ISO 179-1:2010 standard. Unnotched and notched specimens were placed in a Charpy test equipment Instron Ceast 5.5 Resil by Ceast S.p.a. (Pianezza, Italy). The equipment was used to measure the energy absorbed during the impact.

## 3. Results and Discussion

### 3.1. Melt Flow Index of the Obtained Blends

**Figure 1:**
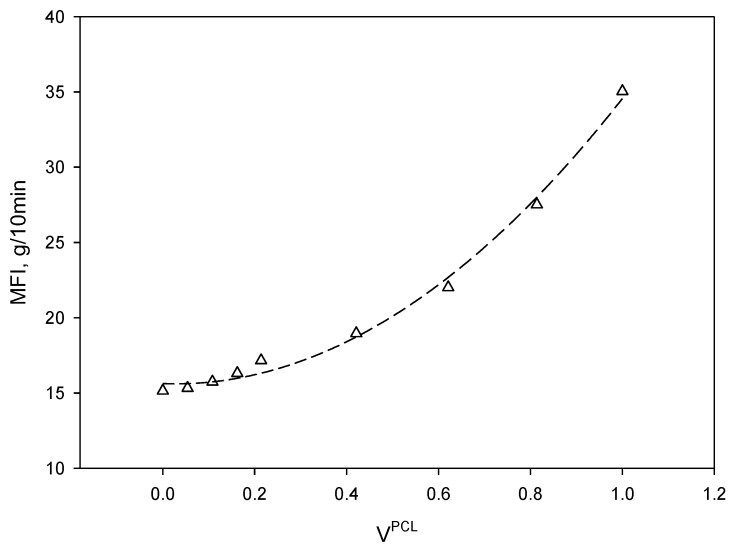
Evolution of melt flow index (MFI) as the amount of PCL was increased. Dashed line corresponds to a second order polynomial regression.

Injection molding conditions (temperature and pressure) were modified depending on the melt flow index (MFI) of the different materials. As stated above, MFI was performed at 210 °C and 2.16 kg of weight. Figure 1 shows the evolution of MFI as function of the volume fraction of PCL (V^PCL^).

As it is possible to see, the evolution of MFI followed a second order polynomial behavior with a correlation factor R^2^ of 0.9943. The MFI of PCL was about 2.32 times higher than PLA under the working conditions, indicating that PCL may flow better than PLA during injection molding, although it can generate burr during the mold filling. For this reason, the injection pressure was decreased as the amount of PCL was increased for the preparation of the standard specimens for mechanical testing.

### 3.2. Tensile Characteristics of the Obtained Blends: Tensile Strength and Young’s Modulus

Neat PLA exhibited a low strain at break, which is typical from glassy polymers, and which limits its use to low deformation applications [32,66]. On the other hand, PCL allows high deformation without breaking, with a high strain at break [53,55,67]. In addition to this, the tensile strength of PLA is significantly higher than in the case of PCL. Indeed, PCL exhibits lower mechanical properties than polypropylene (PP) or high-density polyethylene (HDPE), while PLA can be considered as a commodity polymer with properties near to those considered as engineering polymers. Table 2 shows the experimental values of the tensile strength of the blends (σ_t_^B^), their Young’s modulus (E_t_^B^) and their strain at maximum stress and at break (ε_t_^B^).

**Figure 2:**
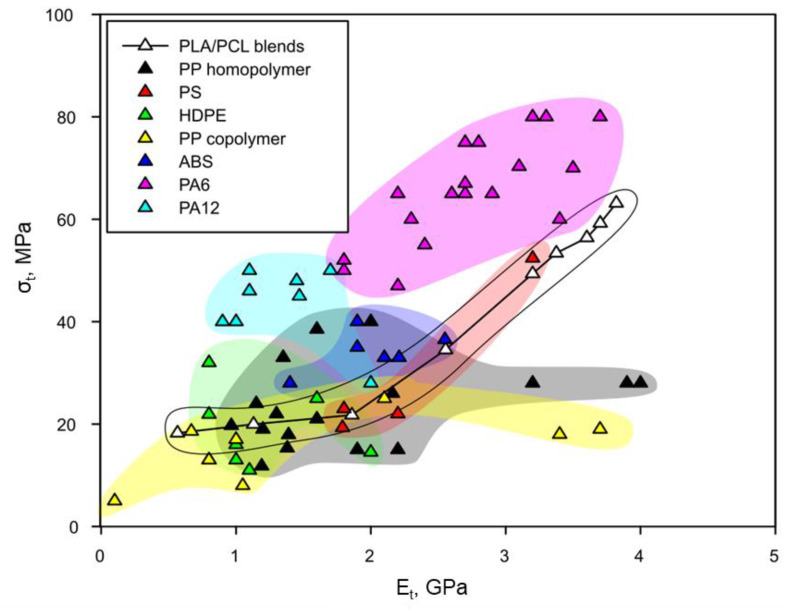
Ashby plot for tensile strength (σ) against Young’s modulus (E) for the obtained blends and compared to other commodity polymers.

PLA exhibited a tensile strength of 63.13 MPa, while, in the case of PCL, this property accounted for 18.25 MPa. The obtained blends exhibited values in the range comprised by the two neat matrices and, as expected, tensile strength decreased as the volume fraction of PCL was increased. A similar behavior was observed in the case of Young’s modulus, which ranged from 3.82 GPa (PLA) to 0.56 GPa (PCL). In the case of elongation at break, it was found that blends containing more than 20 wt% of PCL (V^PCL^ of 0.214) did not break within the tested range and the elongation at maximum stress was significantly increased by the addition of PCL. For this PCL content, the decrease on the tensile strength accounted for a 22%, being of the same magnitude than those reported by other authors when no coupling is induced between the two phases [55,67]. The main advantage of combining PLA and PCL is the wide range of properties that can be obtained. This spectrum provides to the obtained materials the suitability to be used in several applications where other commodity oil-based polymers are currently used [68]. For comparison purposes, Figure 2 shows the tensile strength of the obtained blends as function of their Young’s modulus compared to commercially available data of other commodity polymers. Specific data of commodity polymers can be accessed in the Supplementary Materials (Tables S1–S7).

PLA/PCL blends exhibited a wide region in the diagram. As expected, those blends containing higher amount of PLA exhibited higher tensile strength and Young’s modulus. Concretely, this high PLA content blends exhibited significantly higher tensile strength for a certain Young’s modulus than some polypropylene (PP) homopolymer and copolymer. PLA/PCL blends could perfectly represent a strong alternative to polyolefin such as PP or high-density polyethylene (HDPE), as it is clear that a wider range of properties can be obtained [32,69]. HDPE showed almost a circular area with Young’s modulus in the range from 0.8 to 2.0 GPa and tensile strengths in the range from 15 to 30 MPa. PLA/PCL blends, at higher PCL contents than 40 wt%, were found in a mid-zone, where it is possible to find HDPE matrices with higher or lower tensile strength for a similar Young’s modulus and vice versa. In this region, PLA/PCL blends are partially capable to substitute HDPE matrices. Materials with lower PCL content exhibited higher tensile strength and Young’s modulus than HDPE, being of importance due to the high amount of HDPE that is currently used in the industry [1]. In general, polyamides (both PA6 and PA12) were found to be more competitive than PLA/PCL blends, especially in the case of PA6. For a specific range of Young’s modulus (i.e., 2–3.5 GPa), PA6 exhibited significantly higher strength than PLA/PCL blends. In the range of 1 to 2 GPa, PA12 also was found to be stronger than PLA/PCL blends. Something interesting is that PLA/PCL blends almost covered all the area of polystyrene (PS) in the Ashby plot. This overlapping between the PLA/PCL spectrum and the PS area indicates that the obtained blends could be potential PS substitutes, at least in strength and modulus terms. Finally, in the case of acrylonitrile butadiene styrene (ABS), for Young’s modulus comprised between 1.5 and 2.7 GPa (40–80 wt% PCL in PLA/PCL blends), PLA/PCL exhibited slightly lower tensile strength but values were significantly improved at higher PCL contents.

**Figure 3:**
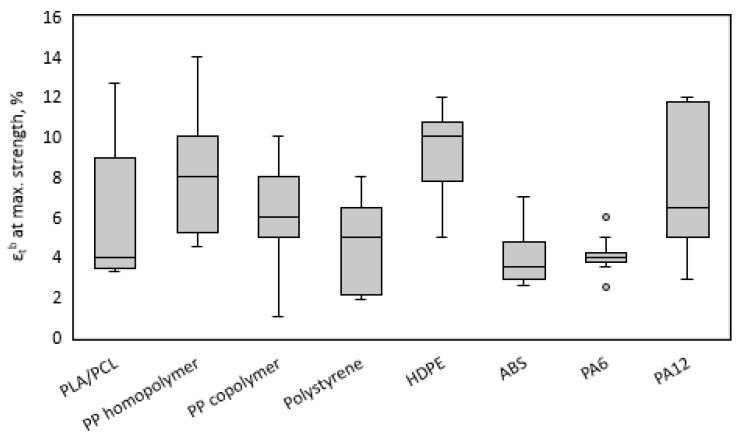
Box and whiskers chart of strain at maximum strength of the obtained PLA/PCL blends compared to other commodity polymers.

Regarding the strain at maximum strength, Figure 3 shows how the PLA/PCL blends compare to commercially available polymers.

PLA/PCL blends exhibited a wide spectrum of strains at maximum strength, a fact that has been previously reported and studied [53,70]. The lowest values of strain were found to those blends with higher contents of PLA. This wide spectrum reveals the versatility of PLA/PCL blends to be used in many applications, apparently including those where the rest of the commodities from Figure 3 can be of use. In fact, the incorporation of PCL into the PLA matrix was proposed to overcome the limitation of the low strain at maximum strength and especially at break of PLA. Only HDPE and PA12 seemed to place their strains at maximum strength outside above the most probable range exhibited by the proposed blends. In any case, PLA/PCL blends showed strains in line with commercial materials, adding another factor supporting their commercial relevance and usefulness.

**Figure 4:**
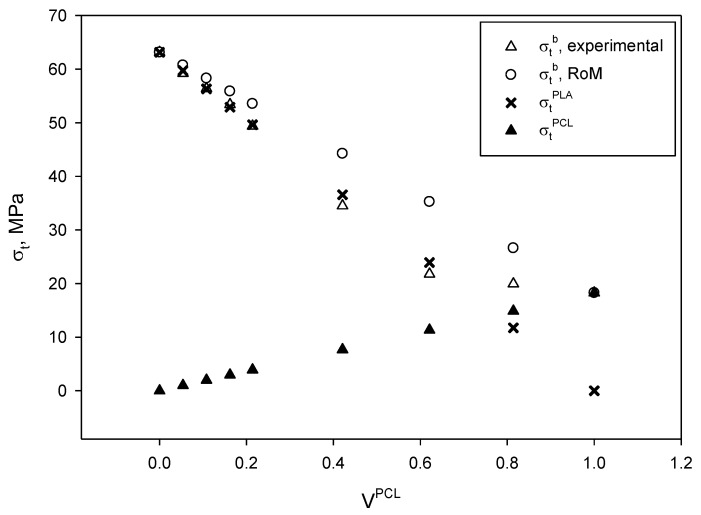
Evolution of tensile strength of PLA/PCL blends as PCL fraction was increased, both experimental and results from the rule of mixtures (RoM) and contributions of each constituent.

The use of different micromechanical models can be interesting in order to understand the interactions between the constituents of the blend, as well as which is the contribution of each phase to their tensile strength. Figure 4 shows the evolution of the tensile strength of the PLA/PCL blends as function of the volume fraction of PCL.

As stated above, the experimental tensile strength of the obtained blends ranged from 63.13 to 18.25 MPa, being the tensile strength of neat PLA and PCL, respectively. The evolution of the tensile strength did not evolve linearly as function of the PCL content and two regions were identified. On the one hand, for blends containing from 0 to 60 wt% of PCL, the tensile strength decreased following a linear tendency and its regression returned a slope of −67.2 for a 0.998 correlation factor (R^2^). On the other hand, those blends containing higher amounts of PCL than 60 wt% exhibited also a decreasing linear tendency, but the slope accounted for −9.3 for a 0.999 correlation factor (R^2^). In addition, this behavior of PLA/PCL blends has been previously reported for increasing amounts of PCL [71,72]. A perfect blend is expected to return a tensile strength that may be predicted by the rule of mixtures (RoM):

(1) $$\sigma_{t}^{B}=\sigma_{t}^{PCL}\times V^{PCL}+\sigma_{t}^{PLA}\times \left(1-V^{PCL}\right),$$

where $\sigma_{t}^{B}$, $\sigma_{t}^{PCL}$, and $\sigma_{t}^{PLA}$ are the theoretical tensile strength of the blend and the experimental tensile strengths of PCL and PLA, respectively. As stated above, $V^{PCL}$ is the volume fraction of PCL within the blend.

Figure 4 shows the expected tensile strengths of the blends compared to the experimental values, as well as the contributions of the PCL and PLA matrices for each PCL content. The experimental values are lower than those predicted by the RoM, but this was totally expected as the model assumes ideal conditions. The figure also shows that the theoretical contribution of PLA was of the same magnitude than the tensile strength of the blends at low and moderate PCL contents, while, in the second region identified, the tensile strength of the blends results from the sum of the individual contributions. 

Another reason for the evolution of the tensile strength of the blends can be due to an overestimation of the contribution of the PCL constituent. In fact, as previously discussed, the strain at break and at maximum strength of the blends increased with the amount of PCL. Thus, the contribution of the constituents will coincide with the stress of the matrix at the strain at maximum strength of the blend. These strains will be always higher than the strain at maximum strength of PLA. Thus, the relative contribution of PLA to the tensile strength of the blend will be always 100%. On the other hand, the strain at maximum strength of PCL is higher than in the case of PLA/PCL blends, overestimating thus its contribution to the resulting material. Nonetheless, this overestimation will decrease as the amount of PCL is increased. For all the above, the RoM is expected to overestimate the tensile strength of the blends.

**Figure 5:**
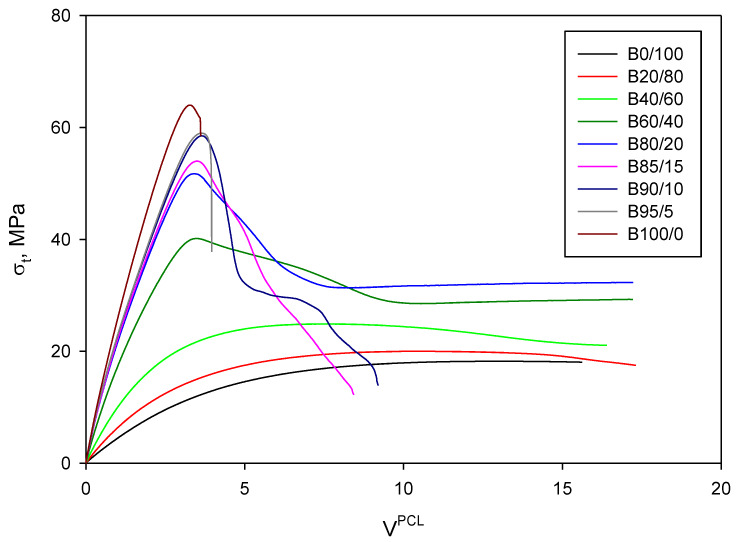
Stress-strain curves of the PLA/PCL blends and PLA and PCL matrices.

A more sophisticated and elaborated version of a rule of mixtures, based on the rule used for short-fiber reinforced composites, replaces the strength of the phases ($\sigma_{t}^{PCL}$ and $\sigma_{t}^{PLA}$) by their contribution at the strains of the blends ($\sigma_{t}^{PCL*}$ and $\sigma_{t}^{PLA*}$). This updated model is provided by Equation (2) and data can be obtained from the stress-strain curves of the different materials (Figure 5).

(2) $$\sigma_{t}^{B}=\sigma_{t}^{PCL*}\times V^{PCL}+\sigma_{t}^{PLA*}\times \left(1-V^{PCL}\right),$$

The polynomial curves to compute the contributions of each material can be used (Equations (3) and (4)).

(3) $$\sigma_{t}^{PLA*}=\{\begin{array}{ll} -0.4483{\left(\varepsilon_{t}\right)}^{4}+2.6882{\left(\varepsilon_{t}\right)}^{3}-7.4228{\left(\varepsilon_{t}\right)}^{2}+30.839\varepsilon_{t}+0.204 & if\;0\leq \varepsilon_{t}\leq 3.27\\ 63.13 & if\;3.27\leq \varepsilon_{t}\leq \varepsilon_{t}^{PLA} \end{array},$$

(4) $$\sigma_{t}^{PCL*}=-0.0005{\left(\varepsilon_{t}\right)}^{4}+0.0271{\left(\varepsilon_{t}\right)}^{3}-0.5355{\left(\varepsilon_{t}\right)}^{2}+4.9756\varepsilon_{t}-1\;\;if\;\;0\leq \varepsilon_{t}\leq 12.65,$$

where $\varepsilon_{t}$, in this case, is the strain at maximum strength of the blend to be evaluated. Equation (1) presents two main limitations compared to Equation (2). While Equation (1) provides the theoretical tensile strength of the blend from the experimental values of each constituent, Equation (2) has, as input data, the contributions that can be calculated from Equations (3) and (4), providing a more accurate result. This fact is relevant, since the expected strain of the blend must be evaluated and it is well known that it does not evolve linearly with the amount of filler or reinforcement. Additionally, PLA exhibits a fragile behavior and all the obtained blends presented higher experimental strains. Thus, according to the new model, the contribution of PLA should be obtained for higher strains than the obtained for PLA. In this sense, authors decided that PLA contributed with its maximum strength. Table 3 shows the obtained values from Equation (2), considering Equations (3) and (4), compared to those obtained with Equation (1).

While Equation (2) provides values with a lower error from the experimental data than Equation (1), the obtained values are still far from reality. Considering the shape of the evolution of tensile strength as PCL amount was increased, especially in the first section of the curve (0–60 wt% PCL), it becomes apparent that there is a notable contribution of PLA. In fact, the contributions of PLA to the tensile strength accounted for similar values than the resulting value for the blends in this range. Thus, the contribution of PCL in this region seems to be almost null or merely symbolic. In the second section of the curve, the combined contribution of the phases does not correspond to a Voigt model, where all the phases are under the same deformation and oppose this deformation with its strength. However, it could be considered that the blends were following the Reuss model, which assumes that the phases are under the same stress and contribute to the blend with its ability to deform under load [73,74,75]. A possible equation that may explain this behavior is shown in Equation (5).

(5) $$\sigma_{t}^{B}=\left(\sigma_{t}^{PLA}\cdot V^{PLA}\right)+\left[V^{PCL}\left(\frac{\sigma_{t}^{PLA}\cdot \sigma_{t}^{PCL}}{\sigma_{t}^{PLA}\cdot V^{PCL}+\sigma_{t}^{PCL}\cdot V^{PLA}}\right)\right],$$

**Figure 6:**
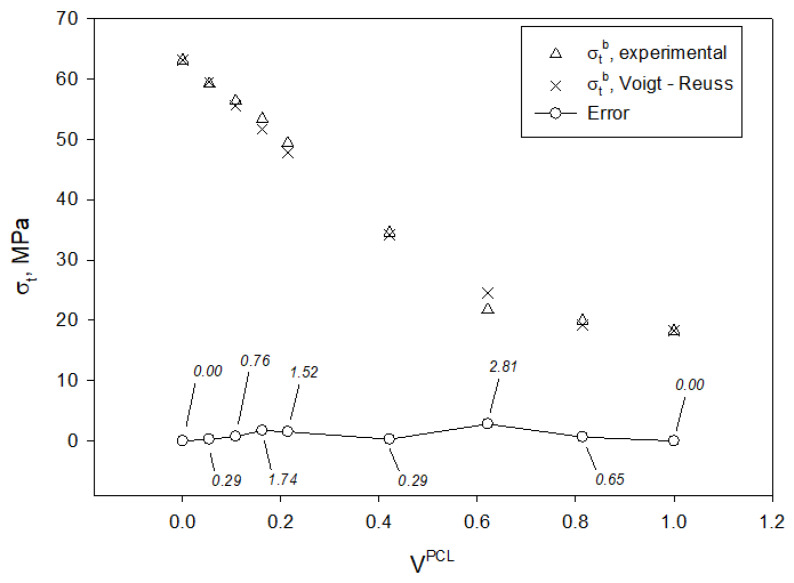
Experimental tensile strength of the PLA/PCL blends compared to the computed values through Voigt-Reuss model.

The equation, in the first term, accounts for the PLA answering to the initial deformations and providing all its strengthening capability. The second term accounts for both phases being under the same deformations until fracture. Figure 6 shows the comparison between the computed tensile strength of the PLA/PCL blends through Equation (5) and the experimental values.

The obtained values through the Voigt-Reuss model were more accurate compared to the experimental values of the tensile strength of the PLA/PCL blends. From this comparison, several details on the interactions between the phases can be assumed. Apparently, PLA contributes to the tensile strength of the blend with its full strength and strengthening capacity, especially for strains comprised in the range of zero to the strain at maximum stress of PLA. This can be clearly observed in the stress-strain curves (Figure 5), where the stresses of the different blends are noticeably high within this range. In terms of contribution to the tensile strength of the blends, PCL acts as a filler and, in addition, its interphase with PLA is weak according to micromechanical modelling, since the individual contribution of PLA reveals that there might be few chemical interactions between both phases [60].

**Figure 7:**
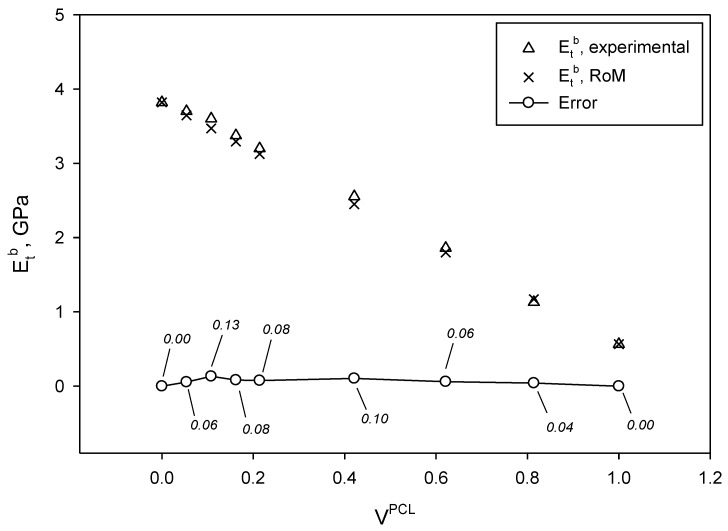
Experimental Young’s modulus of the PLA/PCL blends compared to the computed values through RoM.

In Table 2, values of Young’s modulus are also provided. Young’s modulus of the obtained blends was also modelled using the RoM (Equation (6)) and the obtained values were compared to the experimental data (Figure 7).

As it is possible to see, the Young’s modulus of the blends decreased following a linear tendency with the PCL contents (R^2^ = 0.998). A linear tendency is usually attributed to good dispersion of the phases, but not necessarily good interphase. In fact, the Young’s modulus in composite materials and blends has been extensively reported to be the result of the sum of contributions of each constituent. Equation (6) reveals this sum of contributions.

(6) $$E_{t}^{B}=E_{t}^{PCL}\times V^{PCL}+E_{t}^{PLA}\times \left(1-V^{PCL}\right),$$

where $E_{t}^{B}$, $E_{t}^{PCL}$, and $E_{t}^{PLA}$ are the theoretical Young’s modulus of the blend and the experimental value of PCL and PLA, respectively.

The computed values of Young’s modulus through the RoM were slightly higher than those experimentally obtained, but within the range provided by the standard deviation at each PCL content (Table 2). Although Young’s modulus has been already compared with other commodity polymers in the Ashby plot from Figure 2, it must be mentioned that blends containing up to 60 wt% of PCL exhibited higher Young’s modulus than PP, which usually accounts for 1.5 GPa [76]. Other commodities, such as PA11, exhibits Young’s modulus in the range of 1.3–1.4 GPa, while HDPE can account for 1 GPa [77,78].

### 3.3. Impact Properties of the Obtained Blends

**Figure 8:**
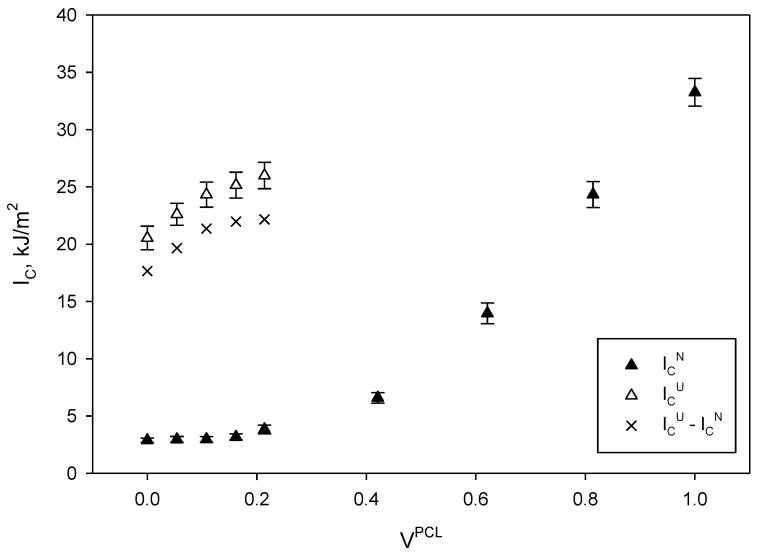
Impact resilience of Charpy notched and unnotched impact strengths of the PLA/PCL blends and fracture creation energy (I_C_^U^−I_C_^N^).

One of the main advantages of blending PCL with PLA, apart from the wide range of properties that can be obtained, with potential opportunities in the commodity markets, is the ability of PCL to improve PLA’s toughness. Toughness is one of the main limitations of PLA, mainly due to its low strain at break and impact strength [48,60,79]. In the previous section, the effect of PCL on tensile properties has been evaluated, concluding that the incorporation of moderate amounts of PCL can have a great impact on the ability of PLA to stand high deformations without breaking. At the same time, the mechanical properties of the blends remain comparable or even better than some commodity polymers such as polypropylene or high-density polyethylene. In this section, the effect of PCL in PLA/PCL blends on their impact strength is evaluated, both for notched (I_C_^N^) and unnotched (I_C_^U^) specimens (Figure 8).

At a glance, both notched and unnotched impact strengths increased with PCL content. The increase was more noticeable in the case of unnotched specimens, where for PCL contents higher than 0.4 the energy needed to break such specimens was higher than the produced by the pendulum.

Impact strength tests are useful to measure two different phenomena. On the one hand, the energy required to create a fracture, and on the other, the energy required to propagate such fracture [80,81]. Therefore, in the case of PLA/PCL blends, the total energy devoted to break a specimen can be explained with Equation (7).

(7) $$w=w_{i}+w_{PLA}+w_{PCL}+w_{B},$$

where *w* is the total amount of energy required to break the specimen. The terms *w_i_*, *w_PLA_*, and *w_PCL_* are the energy needed to create a fracture and propagate it along the PLA and PCL, respectively. The term *w_B_* refers to complex interactions in the interphase between the blend constituents. In the case of notched specimens, the energy devoted to the creation of the fracture can be neglected, as such fracture is already provided by the notch. Thus, this energy will approximately be the difference between the impact strength of unnotched and notched specimens (I_C_^U^−I_C_^N^ in Figure 8).

The PLA/PCL blends B100/0, B95/5, B90/10, and B80/20 (from 0 to 0.2 of V^PCL^ approximately) showed similar impact strength in the case of notched specimens, being almost the same than neat PLA. This is in consonance with the obtained results of tensile strength and modulus, where, for low PCL contents, it was found that PLA was the main contributor. Since at this low-moderate PCL contents the contribution of the last is minor, the crack propagation will surely occur through the PLA and PLA/PCL interphase. The slight increase on the impact strength may come from the increasing area represented by the interphase and in less extent from the contribution of PCL This brittle region of the curve coincides with those materials that broke under unnotched conditions. Considering that the impact strength of unnotched specimens increased, the presence of PCL certainly improved the energy required to create a fracture, indicating that PCL absorbed most of the energy of the pendulum, as shown by the slope of the curve. Other authors found that the incorporation of moderate amounts of PCL into PLA matrices did not have a significant toughening effect. López-Rodríguez et al. (2006) found that the increase on the strain at break accounted for 1.3% when 20 wt% of PCL was incorporated, being an increase of the same magnitude than in the present study [65]. Other authors, such is the case of a recent study published by Kassos et al. (2019), indicated that the impact strength of the PLA/PCL blends containing 30 wt% of PCL accounted for almost twice the strength of PLA, but the evolution of this property as PCL content was increased followed a strange tendency [63]. Ostanfika et al. (2015) obtained higher impact strength for the blend B80/20, but this was the result of a methodic treatment of the morphologies of the blend constituents by means of the incorporation of TiO_2_ into the blends. In fact, the authors obtained higher strain than in the case of neat PCL [79]. 

At higher PCL contents, unfortunately the pendulum was not able to provide enough energy to break the specimens and the analysis remains focused on the notched ones. Qualitatively, the unnotched strength of the blends is expected to be enhanced with increasing amounts of PCL, mainly due to the energy needed to originate the fracture. Taking into account the stress-strain curve of PCL from Figure 5, it can be observed that it is significantly higher than the area below the PLA stress-strain curve. To the same extent, notched specimens exhibited an increasing tendency of impact strength with increasing amounts of PCL, indicating a transition from brittle to ductile fracture. Presumably, the fracture may propagate through the PCL matrix, involving deformations, and breaking PLA areas or PLA/PCL interphase, where the weakest regions can be found. Nonetheless, PLA and PCL cannot be considered as incompatible at all, but the particle size of PCL within the PLA matrix, as well as their crystallinity, play a key role on the resulting toughness of the blends [60]. Other authors have reported the need of adding compatibilizers into the PLA/PCL blends, based on polyethylene glycol, maleic anhydride, dicumyl peroxide or others, but toughness was not significantly improved after their addition, making unjustifiable their use for enhancing toughness of materials [61,64,67].

## 4. Conclusions

PLA/PCL blends have been successfully prepared by means of batch extrusion and processed in an injection molding equipment. From the obtained results, it can be concluded that the obtained blends are potential substitutes of several oil-based commodity plastics, in terms of tensile strength, Young’s modulus and strain. The incorporation of moderate amounts of PCL into the PLA matrix significantly decreased the tensile strength, in about 22% for a 20 wt% addition. In the region of 0 to 40 wt% of PCL content, the PLA seemed to govern the mechanical performance of the blends, but at higher dosages, the evolution of the tensile strength was different. The tensile strength behavior differed significantly from the rule of mixtures, but it was successfully adjusted to the combination between Voigt and Reuss models. On the other hand, the Young’s modulus evolved linearly with increasing amounts of PCL, indicating a good dispersion of the constituents of the blend. PCL was found to provide a good toughening effect to PLA, increasing the required energy to propagate and generate the fracture, especially for contents higher than 20 wt%. In fact, PLA/PCL containing more than 20 wt% of PCL did not break during Charpy testing. Overall, it was found that the obtained PLA/PCL blends, because of their bio-based/biodegradable nature, can constitute a strong and environmentally-friendly alternative to oil-based commodity materials, especially polypropylene and high-density polyethylene, but also others such as polystyrene.

## Figures and Tables

**Table 1:** Experimental batch of the present study.

Sample	Polylactic Acid (PLA) (wt%)	Polycaprolactone (PCL) (wt%)	V^PCL^
B100/0	100	0	0.000
B95/5	95	5	0.054
B90/10	90	10	0.108
B85/15	85	15	0.162
B80/20	80	20	0.214
B60/40	60	40	0.421
B40/60	40	60	0.621
B20/80	20	80	0.814
B0/100	0	100	1.000

**Table 2:** Tensile strength, Young’s modulus and strains at break and at maximum stress of the PLA/PCL blends.

V^PCL^	σ_t_^B^ (MPa)	E_t_^B^ (GPa)	ε_t_^B^ at Break (%)	ε_t_^B^ at Max. Stress (%)
0.000	63.13 ± 2.32	3.82 ± 0.21	3.68 ± 0.12	3.27 ± 0.09
0.054	59.22 ± 2.03	3.70 ± 0.13	4.79 ± 0.21	3.39 ± 0.16
0.108	56.38 ± 2.96	3.60 ± 0.26	6.86 ± 0.32	3.64 ± 0.23
0.162	53.39 ± 3.01	3.38 ± 0.27	10.07 ± 0.29	3.50 ± 0.28
0.214	49.37 ± 1.86	3.20 ± 0.19	15.32 ± 0.56	4.01 ± 0.24
0.421	34.51 ± 2.56	2.55 ± 0.09	-	5.63 ± 0.34
0.621	21.77 ± 1.79	1.86 ± 0.11	-	7.56 ± 0.45
0.814	19.95 ± 0.96	1.13 ± 0.05	-	10.39 ± 0.51
1.000	18.25 ± 1.03	0.56 ± 0.07	-	12.65 ± 0.55

**Table 3:** Theoretical tensile strength of the blends computed with different versions of RoM compared to experimental values.

V^PCL^	σ_t_^B^ exp. (MPa)	σ_t_^B^ Equation (1) (MPa)	σ_t_^B^ Equation (2) (MPa)	σ_t_^B^−σ_t_^PCL*^ (MPa) ^1^
0.000	63.13 ± 2.32	63.13	63.13	63.13
0.054	59.22 ± 2.03	60.71	60.30	59.72
0.108	56.38 ± 2.96	58.28	57.53	56.31
0.162	53.39 ± 3.01	55.86	54.68	52.90
0.214	49.37 ± 1.86	53.53	52.18	49.62
0.421	34.51 ± 2.56	44.24	42.60	36.55
0.621	21.77 ± 1.79	35.26	33.92	23.93
0.814	19.95 ± 0.96	26.60	25.95	11.74
1.000	18.25 ± 1.03	18.25	18.25	0.00

^1^ σ_t_^B^ experimental.

## Supplementary Materials

The following are available online at https://www.mdpi.com/1996-1944/13/11/2655/s1, Table S1: List of polypropylene homopolymer, Table S2: List of polypropylene copolymer, Table S3: List of high density polyethylene, Table S4: List of acrylonitrile butadiene styrene, Table S5: List of polystyrene, Table S6: List of polyamide 12, Table S7: List of polyamide 6.
