# Supplementary material for: Polylactic Acid/Polycaprolactone Blends: On the Path to Circular Economy, Substituting Single-Use Commodity Plastic Products
Source: Materials (Basel). 2020 Jun 10;13(11):2655. doi: 10.3390/ma13112655 (PMC7321633; doi:10.3390/ma13112655)
Supplement: Supplementary file 1 [file materials-13-02655-s001.pdf]

materials

MDPI

Article

Polylactic Acid/Polycaprolactone Blends: On the Path to Circular Economy, Substituting Single-Use Commodity Plastic Products

Marc Delgado-Aguilar [1,2,*], Rita Puig [1], Ilija Sazdovski [2] and Pere Fullana-i-Palmer [2]

1 ABBU research group. Department of Computer Science and Industrial Engineering, Universitat de Lleida (UdL), Pla de la Massa 8, 08700 Igualada, Spain; rita.puig@udl.cat
2 UNESCO Chair in Life Cycle and Climate Change ESCI-UPF, Universitat Pompeu Fabra. Passeig Pujades 1, 08003 Barcelona, Spain; ilija.sazdovski@esci.upf.edu (I.S.); pere.fullana@esci.upf.edu (P.F.P.)
* Correspondence: mda13@alumnes.udl.cat

Received: 20 May 2020; Accepted: 8 June 2020; Published: date

Table S1. List of polypropylene homopolymer.

Polypropylene, Homopolymer		
**Producer**	**Product Reference**	**Reference**
Aclo Accucomp	HPO306 Homopolymer	http://matweb.com/search/DataSheet.aspx?MatGUID=ec6a423060864423a465d50f86d69d4c
Aclo Accucomp	HPO309L Homopolymer	http://matweb.com/search/DataSheet.aspx?MatGUID=c1892f6331d444e3930b183a8307b3b0
Borealis	Daploy WB140HMS	http://matweb.com/search/DataSheet.aspx?MatGUID=2e64444aa5b24dabade4ac0747421221
Dow/DuPont	D114.01	http://matweb.com/search/DataSheet.aspx?MatGUID=3fad3d1abcb54ed79df43ee103cb2327&ckck=1
Dow/DuPont	INSPIRE™ 112 Performance Polymer	http://matweb.com/search/DataSheet.aspx?MatGUID=70bb049f3cc84ebca81c0c0731480481
Lyondell Basell	EA600P	http://matweb.com/search/DataSheet.aspx?MatGUID=5f42020a8bb043d6a69ad979439c547a
Addiplast	Addilene H 652	http://matweb.com/search/DataSheet.aspx?MatGUID=1faa76ca2fda45d18799e3001a2a6759
Addiplast	Addilene H 242 M40	http://matweb.com/search/DataSheet.aspx?MatGUID=49d97633191f45a4b0ddd4b1c7e55673
Addiplast	Addilene H 442 M30	http://matweb.com/search/DataSheet.aspx?MatGUID=752a30ace126432da449f2e905cc444f
Addiplast	Addilene H 520 M40	http://matweb.com/search/DataSheet.aspx?MatGUID=3dc0dae7784842bfbfe909cd725ee922
Lati Industria Termoplastici S.P.A	Latene® 11 MDT05-01	http://matweb.com/search/DataSheet.aspx?MatGUID=1f740441651f423fb14ca4732ab94583
Lati Industria Termoplastici S.P.A	Latigray 52/11-01 CX/45	http://matweb.com/search/DataSheet.aspx?MatGUID=6c2157f3341a495994199797e6e7d812

Manifattura Cattaneo S.P.A	PP H Polypropylene	http://matweb.com/search/DataSheet.aspx?MatGUID=a10aa0acf11e4995a41229df7194d67e
Ineos	H25E-00	http://matweb.com/search/DataSheet.aspx?MatGUID=87734ccab7dd4bcf8a31ccd20bd851b0
LNP (GE Plastics Company)	Colorcomp® M-100 HS GN4-52	http://matweb.com/search/DataSheet.aspx?MatGUID=33daba79f3b54c02b8d0c37631123b12
LNP (GE Plastics Company)	Stat-Loy® M-Polypropylene	http://matweb.com/search/DataSheet.aspx?MatGUID=d0f2f19417d940ef83ce850fd7f43f9f
Trinseo	INSPIRE™ TF3800	http://matweb.com/search/DataSheet.aspx?MatGUID=1c55e00505e74eb9a45b1dd0a4cd12d1

Table S2. List of polypropylene copolymer.

Polypropylene, Copolymer		
**Producer**	**Product Reference**	**Reference**
Aclo Accucomp	CPO407L	http://matweb.com/search/DataSheet.aspx?MatGUID=4bad29c7efea4a2f9c6f0195da2a6353
Aclo Accucomp	CPO406L	http://matweb.com/search/DataSheet.aspx?MatGUID=51236c4a5bb141f3b80b4d824a760e13
Addiplast	Addilene J 220 M40	http://matweb.com/search/DataSheet.aspx?MatGUID=f4fc7c1ce2c848458d8f6e6d468d9ed1
Addiplast	Addilene J 520 M40	http://matweb.com/search/DataSheet.aspx?MatGUID=bdad7169001f43f8b22a6b38d1289165
Ineos	R01A-00	http://matweb.com/search/DataSheet.aspx?MatGUID=c99c6640fd45446aab61e43571dd2c93
Lati Industria Termoplastici S.P.A	Latigray 47/1-01 CX/45	http://matweb.com/search/DataSheet.aspx?MatGUID=f1675053a8b848c193456c800a40ef71
Lati Industria Termoplastici S.P.A	Latimass 47/7-04 D008	http://matweb.com/search/DataSheet.aspx?MatGUID=9ba81777284e46a39a9fb4b067e9361c
Lati Industria Termoplastici S.P.A	Latistat 47/7-03	http://matweb.com/search/DataSheet.aspx?MatGUID=1c8eda36ae6c4bd4a397805d48d5399e

Table S3. List of high density polyethylene.

HDPE		
**Producer**	**Product Reference**	**Reference**
Aclo Accucomp	HD0202L	http://matweb.com/search/DataSheet.aspx?MatGUID=f1755a4ccfda4e0e9d6f406c3f99bfb5
Aclo Accucomp	HD0205L	http://matweb.com/search/DataSheet.aspx?MatGUID=174ecdea264d41f383b65f18376d3ff2
Lyondell Basell	Alathon® M5372	http://matweb.com/search/DataSheet.aspx?MatGUID=4d2ec186ebb14af9a7bbfcc3d7d60c65
Lyondell Basell	Alathon® H4620	http://matweb.com/search/DataSheet.aspx?MatGUID=7100107286ff47f192678fd372498f9c

ExxonMobil Chemical	Paxon™ AS55-003	http://matweb.com/search/DataSheet.aspx?MatGUID=51f0068b4a4145b59e1e3cbe689fa083
Iran Petrochemical Commercial Co.	EX 1 HF 7740F	http://matweb.com/search/DataSheet.aspx?MatGUID=8bcd376dab704fc988c6fb92c69da732
Lati Industria Termoplastici S.P.A	Latistat 45/7-02	http://matweb.com/search/DataSheet.aspx?MatGUID=e68447c7d6a04cbcbf6aed924a7de348
Premix Thermoplastics Inc.	PRE-ELEC® PE 1295	http://matweb.com/search/DataSheet.aspx?MatGUID=cebdc5aba5954488a209eee4e1197209

Table S4. List of acrylonitrile butadiene styrene.

ABS		
**Producer**	**Product Reference**	**Reference**
Aclo Accucomp	ABS802L	http://matweb.com/search/DataSheet.aspx?MatGUID=5fd1721c268c4f69bd5fbf992cd15a8c
Aclo Accucomp	ABS804L	http://matweb.com/search/DataSheet.aspx?MatGUID=26236bd63cf44c869f9a1946cc6b99a2
Aclo Accucomp	ABS801L	http://matweb.com/search/DataSheet.aspx?MatGUID=aae151d3595a4104818b833124ffe303
Aclo Accucomp	ABS803L	http://matweb.com/search/DataSheet.aspx?MatGUID=cb6d1a8ad959465e8e946c14ad46de91
Gehr Plastics	ABS Acrylonitrile Butadiene Styrene	http://matweb.com/search/DataSheet.aspx?MatGUID=83a6bf2414554dffafebd9e0816bfa8c
Ineos	Lustran® M205FC	http://matweb.com/search/DataSheet.aspx?MatGUID=11eadcd4835a444bb121c9a23e672731

Table S5. List of polystyrene.

Polystyrene (PS)		
**Producer**	**Product Reference**	**Reference**
Dow Chemical	AIM® 4800	http://matweb.com/search/DataSheet.aspx?MatGUID=d647848b16104eb0978e26437c1e9100
Americas Syrenics	Styron® 685P	http://matweb.com/search/DataSheet.aspx?MatGUID=3c98cec5c8ca4317877383003b082926
Premix Thermoplastics Inc.	PRE-ELEC® PS 1326	http://matweb.com/search/DataSheet.aspx?MatGUID=c68e697c451640afb35a1067821c580d
Premix Thermoplastics Inc.	PRE-ELEC® PS 1328	http://matweb.com/search/DataSheet.aspx?MatGUID=667d096b99d6488383189a6c141f95bf
Albis Plastics	ALCOM® MED PS 1000 14113	http://matweb.com/search/DataSheet.aspx?MatGUID=cf4d2324d47d49a5b03fcacd39d0db99
Styrolution	Polystyrol 168 N GR 21 PS	http://matweb.com/search/DataSheet.aspx?MatGUID=beea4f52f84b4db39bd1238e76d1af9a

Styrolution	Polystyrol 495 F GR 2 PS	http://matweb.com/search/DataSheet.aspx?MatGUID=9313ea581b2b413fb51580f8f3f2df64
Styrolution	PS 456EB HIPS	http://matweb.com/search/DataSheet.aspx?MatGUID=2af6ee4235464fe5839133dbf0ad29d1
Styrolution	PS 476L HIPS	http://matweb.com/search/DataSheet.aspx?MatGUID=67ccec567039436fa2aaaa8d3f007ee7
Styrolution	PS 495F HIPS	http://matweb.com/search/DataSheet.aspx?MatGUID=15f758d105e648e7bfc5b656945fdbec
Styrolution	PS 576H HIPS	http://matweb.com/search/DataSheet.aspx?MatGUID=030033e525d24b58b21a2736bbc87b3a
Chi Mei	Polyrex® PG-22	http://matweb.com/search/DataSheet.aspx?MatGUID=0aea84ce3e734314bc8be6b3d203f938
Chi Mei	Polyrex® PG-33	http://matweb.com/search/DataSheet.aspx?MatGUID=ff576d11bc6c449ab32a86ffed358f82
Chi Mei	Polyrex® PG-22	
Chi Mei	Polyrex® PG-22	
Chi Mei	Polyrex® PG-22	

Table S6. List of polyamide 12.

**Polyamide 12 (PA12)**		
**Producer**	**Product Reference**	**Reference**
Evonik	VESTAMID® LX9008	http://matweb.com/search/DataSheet.aspx?MatGUID=07adfdc316e14af0a58d3338ae669c53
Evonik	VESTAMID® LX9012	http://matweb.com/search/DataSheet.aspx?MatGUID=1820960db3fe4684b5644333d39c7b2e
Gehr Plastics	PA 12 TR	http://matweb.com/search/DataSheet.aspx?MatGUID=4c337df134b54ca594feab74788c9cee
EMS-Grivory	Grivory® L 20 G grey 9280 PA12	http://matweb.com/search/DataSheet.aspx?MatGUID=da1af78735f94583beb689859cc030ac
EMS-Grivory	Grivory® L 25 NZ ESD PA12	http://matweb.com/search/DataSheet.aspx?MatGUID=75146c49451845a0ab2e4f1cefa69b22
EMS-Grivory	Grivory® L 25 Z PA12-I	http://matweb.com/search/DataSheet.aspx?MatGUID=400ade8581d0492885103ab1852a3cad
EMS-Grivory	Grivory® LM-05 HX nat PA12	http://matweb.com/search/DataSheet.aspx?MatGUID=9252cb6fe4cc442f8a3a00e239bed86e
Sabic	LNP™ LUBRICOMP™ SP003	http://matweb.com/search/DataSheet.aspx?MatGUID=299c207bdc3c45129cd4a2adabe29ba9

Table S7. List of polyamide 6.

**Polyamide 6 (PA6)**		
**Producer**	**Product Reference**	**Reference**

Aclo Accucomp	Nylon 6 NY0700L	http://matweb.com/search/DataSheet.aspx?MatGUID=be55ac610c4a4b12b523379841a346c5
BASF	Ultramid® 8202 PA6	http://matweb.com/search/DataSheet.aspx?MatGUID=d8d83fe59e0b47148639f58bf2642638
BASF	Ultramid® 8253 PA6	http://matweb.com/search/DataSheet.aspx?MatGUID=fd18fb2b0fc84260829dcd49e91f2e6a
DSM	Akulon® FL-LP PA6	http://matweb.com/search/DataSheet.aspx?MatGUID=4256e304c46a427fad5eef1cda98afaf
Grupo Repol	Dinalon® B1S25 PA6	http://matweb.com/search/DataSheet.aspx?MatGUID=2264e87ee1394c8292e1aa62ab483499
Grupo Repol	Dinalon® B1V25 PA6	http://matweb.com/search/DataSheet.aspx?MatGUID=2019d2a466214ce2979c98b7768a72a0
Grupo Repol	Dinalon® B3S25 PA6	http://matweb.com/search/DataSheet.aspx?MatGUID=2fa1cb26afde4dd0a6c5b1b185e76f2a
Lanxess	Durethan® B 30 S FN30 000000	http://matweb.com/search/DataSheet.aspx?MatGUID=8ddee30e62434624af849f9abe45d791
Lanxess	Durethan® B 30 S FN40 000000	http://matweb.com/search/DataSheet.aspx?MatGUID=abf40efae4884cf6b6ed44e5256891df
Lati Industria Termoplastici S.P.A	Latilub 62-10T PA6	http://matweb.com/search/DataSheet.aspx?MatGUID=a49fa6a68d434fc7b220f98b38f112df
Lati Industria Termoplastici S.P.A	Latilub 62-15ST PA6	http://matweb.com/search/DataSheet.aspx?MatGUID=714baacda36147cf90e9ff6c9d4d4f37
Nurel Engineering Polymers	Promyde® B30 P MI PA6	http://matweb.com/search/DataSheet.aspx?MatGUID=bb92de2ebf484f669eb639fb22141999
Nurel Engineering Polymers	Promyde® B30 P2 HI PA6	http://matweb.com/search/DataSheet.aspx?MatGUID=581d628533ef4cb793190742fb26b850
Solvay	TECHNYL® C 50 H2 PA6	http://matweb.com/search/DataSheet.aspx?MatGUID=67290439286b4fc097162adeaec0e31e
Solvay	TECHNYL® C 236SI PA6	http://matweb.com/search/DataSheet.aspx?MatGUID=91cde4bf2aa14daa949539ef8000d14a
Teknor Apex	Chemlon® HY2SM Nylon 6	http://matweb.com/search/DataSheet.aspx?MatGUID=418cd67465f24ebf9d7421908b725a5b
Teknor Apex	Chemlon® MD3G Nylon 6	http://matweb.com/search/DataSheet.aspx?MatGUID=e0162b3559294e9689002d1ea202f22a
Teknor Apex	Chemlon® MD4 Nylon 6	http://matweb.com/search/DataSheet.aspx?MatGUID=d6cce451d7964ef1933ea74f81ca2da0
Ter Hell Plastic Gmbh	Terez® PA6 7750 TK	http://matweb.com/search/DataSheet.aspx?MatGUID=f03b24fb1b7944c7985b29374c05ca16
Tisan	TISLAMID® 6 UNR Nylon 6	http://matweb.com/search/DataSheet.aspx?MatGUID=245a5ba7feb94330b50ac7ed434b952d

Unitika Ltd.	CX-2500 PA6	http://matweb.com/search/DataSheet.aspx?MatGUID=e009e28a28b74fc2be2876870b6b6fa2
